# Radiomics Outperforms Clinical and Radiologic Signs in Predicting Spontaneous Basal Ganglia Hematoma Expansion: A Pilot Study

**DOI:** 10.7759/cureus.37162

**Published:** 2023-04-05

**Authors:** Ali Rezaei, Houman Sotoudeh, Ryan Godwin, Veeranjaneyulu Prattipati, Aparna Singhal, Mahsan Sotoudeh, Manoj Tanwar

**Affiliations:** 1 Radiology, University of Alabama at Birmingham, Birmingham, USA; 2 Anesthesiology and Perioperative Medicine, University of Alabama at Birmingham, Birmingham, USA; 3 Statistics, Azad University of Arak Branch, Arak, IRN

**Keywords:** machine learning, radiomics, hematoma expansion, hematoma, basal ganglia

## Abstract

Prediction of the hematoma expansion (HE) of spontaneous basal ganglia hematoma (SBH) from the first non-contrast CT can result in better management, which has the potential of improving outcomes. This study has been designed to compare the performance of “Radiomics analysis,” “radiology signs,” and “clinical-laboratory data” for this task. We retrospectively reviewed the electronic medical records for clinical, demographic, and laboratory data in patients with SBH. CT images were reviewed for the presence of radiologic signs, including black-hole, blend, swirl, satellite, and island signs. Radiomic features from the SBH on the first brain CT were extracted, and the most predictive features were selected. Different machine learning models were developed based on clinical, laboratory, and radiology signs and selected Radiomic features to predict hematoma expansion (HE). The dataset used for this analysis included 116 patients with SBH. Among different models and different thresholds to define hematoma expansion (10%, 20%, 25%, 33%, 40%, and 50% volume enlargement thresholds), the Random Forest based on 10 selected Radiomic features achieved the best performance (for 25% hematoma enlargement) with an area under the curve (AUC) of 0.9 on the training dataset and 0.89 on the test dataset. The models based on clinical-laboratory and radiology signs had low performance (AUCs about 0.5-0.6).

## Introduction

Intracerebral hemorrhage (ICH) is responsible for about 10% to 20% of all strokes and is associated with higher morbidity and mortality than ischemic strokes [1,2]. ICH yearly occurs in 25 cases per 100,000 population, and the incidence, mortality, and morbidity are higher among Asians compared to the West. About 40% of patients with ICH die within the first month, and only 12%-39% of survivors will achieve long-term functional independence [1,2]. Spontaneous intracerebral hemorrhage (sICH) is described as a non-traumatic hemorrhage without underlying lesions such as a tumor or a vascular abnormality [3]. Spontaneous basal ganglia hemorrhage (sBGH), the most common form of sICH, is associated with high mortality risk, resulting in death or disability in more than 70% of the cases [4]. Multiple factors such as advanced age, poor initial clinical neurological state, a large hematoma, intraventricular spread, and midline shift are independent predictors of poor prognosis [5]. Many authors define cerebral hematoma expansion (HE) as an increased hemorrhage volume of more than 33% or an absolute increase of more than 6 mL on follow-up CT within 24 hours [6]. However, other thresholds have been used by different authors, including a 50% increase in volume [7] and absolute volume enlargement of more than 20 mL [7] or 12.5 mL [8]. HE occurs in about one-third of sBGH patients and is associated with higher mortality [9].

The pathological process of HE is not fully understood. “Avalanche model” hypothesized that the pressure effect of growing initial bleeds compressed the surrounding parenchyma and consecutively resulted in the rupture of multiple peripheral vessels, which ultimately caused hematoma growth and expansion [10]. Continuous rupturing and hemorrhaging of small vessels are the other possible underlying etiology for HE, which can explain the HE in patients with elevated systolic blood pressure [11,12]. Nevertheless, given that the majority of cases with ICH do not have HE, determining patients at risk of expansion is crucial for patient management. Predicting the chance of HE in each patient may improve the patient outcome through early intraventricular shunt placement and recently evolving minimally invasive techniques for evacuating hematoma [13].

So far, different clinical and laboratory features described to be associated with HE include male gender, age older than 85 years old, elevated systolic blood pressure, variation of the systolic blood pressure during admission, anticoagulant and antiplatelet therapy, National Institutes of Health Stroke Scale (NIHSS), and intracerebral hemorrhage (ICH) score, elevated temperature, baseline weight, history of alcohol abuse, and history of cerebral infarction [1]. Spot sign on the arterial phase of CT angiogram (CTA) has a moderate performance (area under the curve (AUC) of 0.74 [14]), while the leakage sign has a sensitivity of 93% and specificity of 90% [15,16]) in HE prediction. However, CTA has limited usage in emergency departments as the first imaging modality [1]. On the first non-contrast CT, “black-hole sign” has moderate performance for this task [14]. On a meta-analysis, the black-hole sign had sensitivity, specificity, and AUC of 30%, 93%, and 0.83, respectively [17]. The “blend sign” sensitivity is between 13% and 42.8%, and its specificity is between 88.5% and 95.5% [18]. The “swirls sign” has a pooled sensitivity and specificity of 50% and 77%, respectively [19]. “Satellite sign” has a sensitivity of 50% and specificity of 71% [20]. Finally, the “island sign” has been suggested as the HE indicator with a sensitivity of 32% and specificity of 92% [21]. Prior authors have shown nine variables (alcohol history, Glasgow Coma Scale score, total serum calcium, blood glucose, international normalized ratio, hematoma shape, hematoma density, volume of hematoma on first computed tomography scan, and presence of intraventricular hemorrhage) as independent predictors of hematoma expansion [22]. Some other factors like hypertension, shorter time to computed tomography (CT), and use of anticoagulation therapy (warfarin) have been identified as independent clinical predictors of HE [23].

With the rapid evolution of artificial intelligence and machine learning, Radiomics techniques have a strong momentum in radiology research. In Radiomics, the region of interest or lesion is first segmented; then, multiple features are extracted from the region of interest. Subsequently, the most predictive features are selected, and machine learning models are trained on these features to predict a clinical target [24]. Radiomics was first developed for oncology; however, its application is not limited to neoplasms [25].

This study was conducted to develop a machine-learning model to predict the expansion of the spontaneous basal ganglia hematoma from the initial brain CT obtained at the time of admission. We use clinical data, including demographics, medical history, physical examination (ICH score, NIHHS score), laboratory tests, radiology signs, and Radiomics features. We compare the performance of “clinical” versus “radiology signs” versus “Radiomics” features in this task. Also, we compare different thresholds to define the HE to find the most suitable threshold for machine learning. This article was previously posted to the medRxiv preprint server on April 27, 2020.

## Materials and methods

This is a retrospective study utilizing clinical, imaging data, and Radiomics to predict expansion in sBGH. Our local university ethical committee approved this retrospective study (IRB-300002728). We reviewed the medical reports from February 2004 to December 2019 and included patients older than 18 years with sBGH on the brain CT at the time of admission and a follow-up brain CT within 24 hours of admission. CT scans were obtained on four Phillips and two Siemens scanners with brain protocols (KV: 120-140, mA: 235-300, slice thickness: 1-5 mm). The patients’ demographic features (age, gender), medical history (history of ICH, ischemic stroke, treatment by antiplatelet, treatment by anti-coagulation, history of hypertension), physical exam information (systolic and diastolic blood pressure, ICH, Glasgow Coma Scale (GCS), and NIHSS scores), and laboratory data (international normalized ratio (INR), blood glucose level) were recorded. Patients with missing data were excluded.

**Figure 1:**
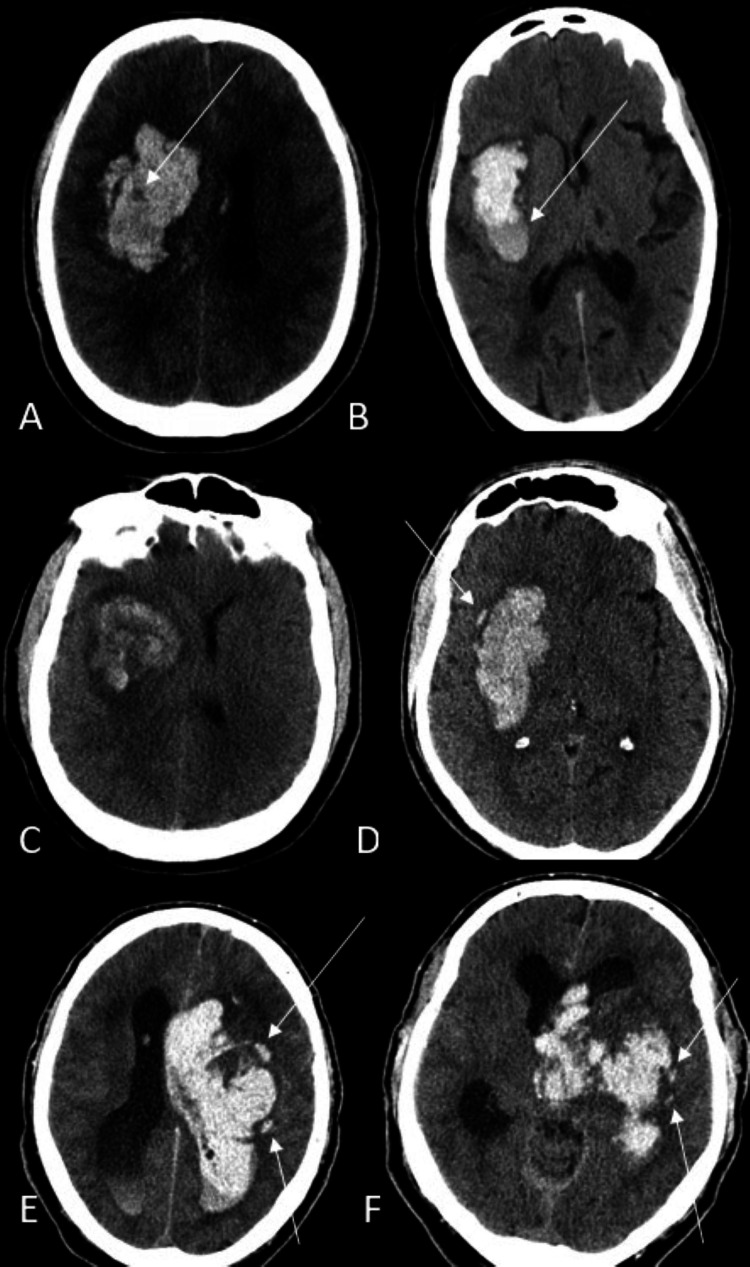
Radiology signs used in this study. A: Black-hole sign (arrow). B: Blend sign (arrow). C: Swirl sign. D: Satellite sign (arrow). E and F: Island sign in one patient (arrows).

The first brain CT at the time of admission was evaluated simultaneously by three neuroradiologists (MT, VP, and HS, four years, 20 years, and 14 years of experience after residency, respectively). The hematomas were scored for the presence of black-hole, blend, swirl, satellite, and island signs by the voting system. Any discrepancies were resolved by discussion. The definition of the radiologist-based signs is summarized in Table 1 and Figures 1A-1E.

**Table 1:** Definition of radiology signs.

Sign	Definition
Black-hole sign (Figure 1A)	A hypoattenuating area within the hyperdense hematoma. There should be more than 28 Hounsfield Unit differences between the two regions, and the relatively hypodense area must be encapsulated by the hyperdense region [14].
Blend sign (Figure 1B)	(1) A blending of the relatively hypoattenuating area with the adjacent hyperdense region within a hematoma; (2) a well-defined margin between the hypodense area and adjacent hyperdense region that is easily identified by the bare eye; (3) the hematoma should have at least an 18 Hounsfield unit difference between the two density regions; and (4) the relatively hypoattenuating area should not be encircled by the hyperdense region [18].
Swirl sign (Figure 1C)	Unclotted fresh blood has a lower density than the clotted blood surrounding it. A low-density region inside hematoma.
Satellite sign (Figure 1D)	A small (maximal transverse diameter <10 mm) hemorrhage totally separate from the main hemorrhage in at least a single slice. The shortest distance between the satellite and the main hemorrhage should be 1-20 mm.
Island sign (Figures 1E, 1F)	(1) ≥3 scattered small-size hematomas all disconnected from the central hematoma or (2) ≥4 small hematomas, some or all of which may connect with the main hematoma. The separated small islands could be round or oval and are detached from the mother hematoma. The small hematomas that connect with the central hematoma (connected islands) could be bubble-like or sprout-like but not lobulated [6].

The sBGHs were manually segmented by a radiologist (AR 10 years of experience) by 3D slicer software, which allows users to delineate hemorrhages in three dimensions. Segmentations were confirmed by the second radiologist (HS). The radiologists were blind to the HE. The volume of hematoma on the baseline and the second CT was calculated based on the 3D segmentation of the hematomas. The difference between the volume on the second and the first CT was considered as HE in percent of enlargement. The Radiomics features of the hematoma on the baseline CT were extracted by the Pyradiomics library.

Different machine learning models (Logistic Regression (LR), Random Forest (RF), Neural Network (NN), Naive Bayes (NB), Support Vector Machine (SVM), k-Nearest Neighbor (KNN), AdaBoost, Gradient Boosting Classifier (GBC), Light Gradient Boosting Machine (LGBM), and Decision Trees (DT)) were developed based on “clinical data” (demographics, laboratory, and physical exam), “Radiology signs,” and Radiomic features. Different targets were tested for the definition of HE (10%, 20%, 25%, 33%, 40%, and 50% volume enlargement).

**Figure 2:**
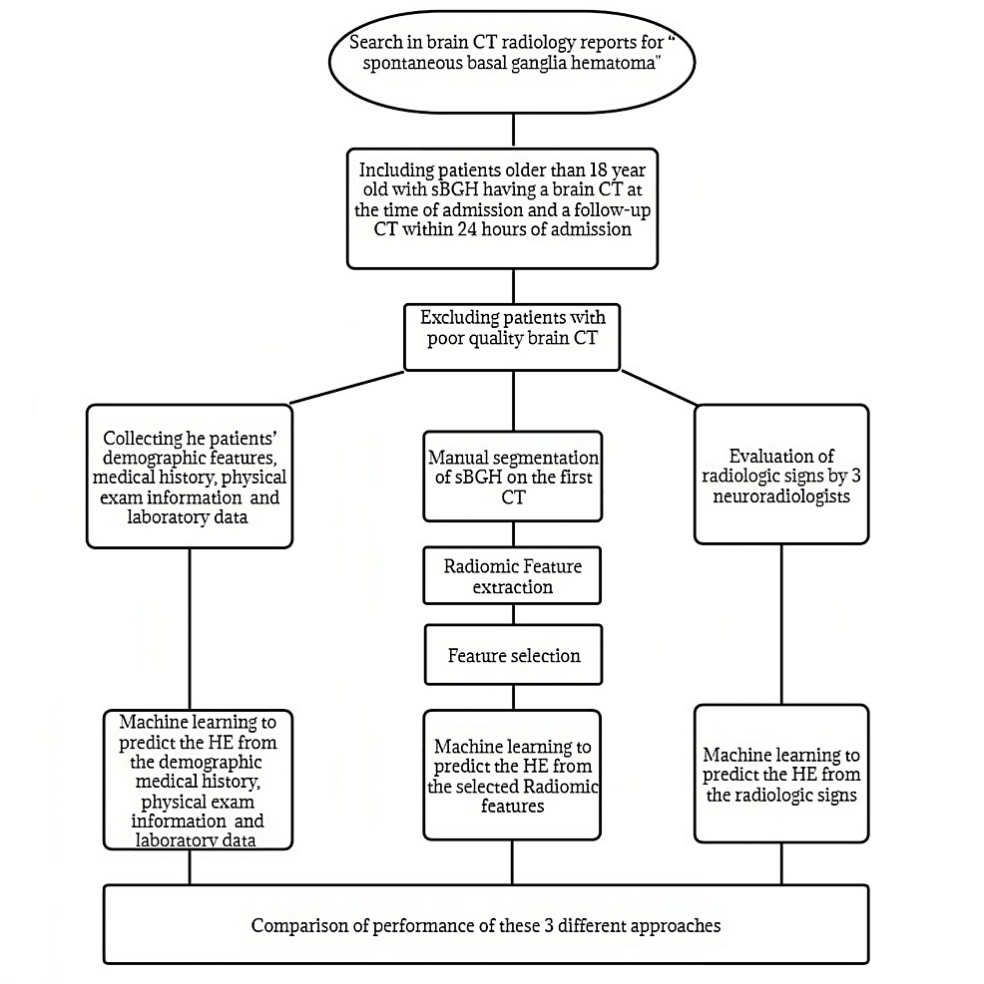
Flowchart of the methodology used in this study. sBGH: spontaneous basal ganglia hemorrhage, HE: hematoma expansion.

To develop the machine learning models, the Radiomic features were first normalized, and the most predictive features were selected by the Least Absolute Shrinkage and Selection Operator (LASSO). For each model, the 10 most predictive features were used (10% of the number of patients in this study). In the last stage, machine learning models were developed on all features (clinical+ radiology signs+Radiomic features) after feature selection (Figure 2).

The performance of each model to predict the HE was reported by the area under the curve (AUC) and accuracy using the 10-fold cross-validation technique. The machine learning was performed utilizing the Orange data mining toolbox in Python [26]. The platform was implemented to run Radiomics analyses on the segmented hemorrhages. All statistical analyses were performed using SPSS version 22 (Chicago, IL: SPSS Inc.). A P-value of 0.05 was considered statistically significant.

## Results

After the implementation of inclusion criteria, 116 patients were included for the final diagnosis (male: 82, female: 34, mean age: 57.4 years). The frequency of HE was 35.3%, 25.8%, 19.8%, 15.5%, 13.7%, and 12.9%, for 10%, 20%, 25%, 33%, 40%, and 50% enlargement of sBGH. The average interval change in volume of the hematoma was +19.8 %. The prevalence of the radiology signs was 7.7%, 12.9%, 23.2%, 16.3%, and 6.8% for the black-hole, blend, swirl, satellite, and island signs, respectively.

The performance of machine learning models based on the “clinical” and “Radiology signs” features was poor and significantly lower than the Radiomic models. The Random Forest-based model on 10 selected Radiomic features achieved the best performance (for 25% hematoma enlargement) with an AUC of 0.9 on the training dataset and 0.89 on the test dataset.

The machine learning models based on a combination of all features (clinical+Radiology signs+Radiomic features) were the same as the Radiomic model because the feature selecting algorithm did not select any feature from the “clinical” and “Radiology sign” features as a predictive one. The only exception was the model to predict a 40% enlargement of hematoma in which “history of hypertension” and “treatment by anti-coagulation” were among the selected features. The results of this study are summarized in Tables 2-5.

**Table 2:** Performance of different machine learning models to predict different thresholds of HE from the “clinical”+“Radiology Signs” at the time of admission. AUC: area under the curve, KNN: k-Nearest Neighbors, NN: Neural Network, GB: Gradient Boosting, SVM: Support Vector Machine, ICH: intracerebral hemorrhage, HE: hematoma expansion, INR: international normalized ratio, NIHSS: National Institutes of Health Stroke Scale.

Definition of HE by volume enlargement	Best model	AUC	Accuracy	Most predictive features
10%	KNN	0.58	63%	INR, systolic blood pressure, ICH score, swirl sign, blood glucose level, diastolic blood pressure, NIHSS score, history of ICH, antiplatelet treatment, age
20%	NN	0.53	66%	INR, swirl sign, NIHSS score, history of ICH, age, ICH score, anticoagulant treatment, history of cerebral infarction, systolic blood pressure, blood glucose level
25%	GB	0.63	75%	Age, INR, history of cerebral infarction, systolic blood pressure, NIHSS score, history of ICH, ICH score, anticoagulant treatment, swirl sign, diastolic blood pressure
33%	SVM	0.57	83%	Systolic blood pressure, history of cerebral infarction, history of ICH, NIHSS score, age, INR, anticoagulant treatment, diastolic blood pressure, ICH score, swirl sign
40%	NN	0.57	81%	Systolic blood pressure, history of ICH, INR, anticoagulant treatment, NIHSS score, ICH score, diastolic blood pressure, age, history of hypertension, history of cerebral infarction
50%	NN	0.62	83%	Systolic blood pressure, INR, history of ICH, anticoagulant treatment, NIHSS score, island sign, diastolic blood pressure, satellite sign, ICH score, age

**Table 3:** The performance of the Radiomic models to predict the different thresholds of HE. Models were ranked on AUC, and comparison was performed on default hyperparameters. Hyperparameters for RF are 100 estimators. ^Performed additional hyperparameter tuning on top model here, which found the optimal parameters were: max depth = 5, max features = 0.99, max leaf nodes = None, with 11 estimators with min sample leaf of 2 and min sample split of 8. *Random Forest was the second-best model. HE: hematoma expansion, AUC: area under the curve, RF: Random Forest, GB: Gradient Boosting, LGBM: Light Gradient Boosting Machine, LR: Logistic Regression, glcm: gray-level co-occurrence matrix, glrlm: gray-level run-length matrix, gldm: gray-level dependence matrix, ngtdm: neighborhood gray-tone difference matrix, glszm: gray level size zone.

Definition of HE by volume enlargement	Best model	AUC (train, test)	Accuracy (train, test)	Selected features
10%	RF	0.759, 0.789	0.708, 0.792	Skewness (first order), variance (first order), median (first order), MeanAbsoluteDeviation (first order), 90 percentile (first order), Autocorrelation (glcm), GrayLevelNonUniformityNormalized (glrlm), SmallDependenceHighGrayLevelEmphasis (gldm), GrayLevelVariance (glrlm), RunEntropy (glrlm)
20%	GB	0.881, 0.815	0.824, 0.875	Variance (first order), strength (ngtdm), JointEntropy (glcm), ClusterTendency (glcm), DependenceVariance (gldm), ShortRunLowGrayLevelEmphasis (glrlm), Kurtosis (first order), Idmn (glcm), skewness (first order), GrayLevelNonUniformityNormalized (glrlm)
25%	RF^	0.9, 0.89	0.804, 0.875	Variance (first order), HighGrayLevelRunEmphasis (glrlm), SmallAreaHighGrayLevelEmphasis (glszm), SumEntropy (glcm), Mean (first order), ZonePercentage (glszm), ZoneEntropy (glszm), SumSquares (glcm), GrayLevelVariance (gldm)
33%	RF	0.858, 0.725	0.827, 0.833	Variance (first order), MeanAbsoluteDeviation (first order), LowGrayLevelRunEmphasis (glrlm), HighGrayLevelZoneEmphasis (glszm), RobustMeanAbsoluteDeviation (first order), HighGrayLevelEmphasis (gldm), ShortRunLowGrayLevelEmphasis (glrlm), GrayLevelVariance (glrlm), HighGrayLevelRunEmphasis (glrlm), ZoneEntropy (glszm)
40%	LGBM	0.711, 0.81	0.837, 0.792	Flatness (first order), history of hypertension, LargeAreaLowGrayLevelEmphasis (glszm), SmallAreaLowGrayLevelEmphasis (glszm), HighGrayLevelRunEmphasis (glrlm), anticoagulation treatment, ClusterShade(glcm), ShortRunLowGrayLevelEmphasis (glrlm), Mean (first order), GrayLevelNonUniformity (glszm)
50%	LR*	0.719, 0.556	0.73, 0.833	LargeAreaEmphasis (glszm), LargeAreaLowGrayLevelEmphasis (glszm), MeshVolume (Shape), LargeAreaHighGrayLevelEmphasis (glszm), SurfaceArea (Shape), LargeDependenceHighGrayLevelEmphasis (gldm), GrayLevelNonUniformity (glelm), GrayLevelNonUniformity (gldm), RunLengthNonUniformity (glrlm), Busyness (ngtdm)

**Table 4:** Performance of different machine learning models to predict different thresholds of HE from the clinical data at the time of admission. HE: hematoma expansion, AUC: area under the curve, KNN: k-Nearest Neighbor, LR: Logistic Regression, GB: Gradient Boosting, SVM: Support Vector Machine, Ada Boost: Adaptive Boosting, ICH: intracerebral hemorrhage, INR: international normalized ratio, NIHSS: National Institutes of Health Stroke Scale.

Definition of HE by volume enlargement	Best model	AUC	Accuracy	Most predictive features
10%	KNN	0.58	63%	INR, systolic blood pressure, ICH score, diastolic blood pressure, NIHSS score, history of ICH, antiplatelet treatment, age, history of cerebral infarction
20%	LR	0.54	61%	INR, NIHSS score, history of ICH, age, ICH score, anticoagulant treatment, history of cerebral infarction, systolic blood pressure, blood glucose level, diastolic blood pressure
25%	GB	0.66	76%	Age, INR, history of cerebral infarction, systolic blood pressure, NIHSS score, history of ICH, ICH score, anticoagulant treatment, diastolic blood pressure, blood glucose level
33%	SVM	0.61	83%	Systolic blood pressure, history of cerebral infarction, history of ICH, NIHSS score, age, INR, anticoagulant treatment, diastolic blood pressure, ICH score, history of hypertension
40%	SVM	0.6	87%	Systolic blood pressure, history of ICH, INR, anticoagulant treatment, NIHSS score, ICH score, diastolic blood pressure, age, history of hypertension, history of cerebral infarction
50%	AdaBoost	0.6	83%	Systolic blood pressure, INR, history of ICH, anticoagulant treatment, NIHSS score, diastolic blood pressure, ICH score, age, history of cerebral infarction, history of hypertension

**Table 5:** Performance of machine learning models to predict HE from five radiology signs of the baseline CT. HE: hematoma expansion, AUC: area under the curve, LR: Logistic Regression, KNN: k- Nearest Neighbor, SVM: Support Vector Machine.

Definition of HE by volume enlargement	Best model	AUC	Accuracy
10%	LR	0.54	63%
20%	KNN	0.5	67%
25%	SVM	0.5	78%
33%	SVM	0.46	83%
40%	SVM	0.46	87%
50%	LR	0.51	87%

## Discussion

In this study, we developed different machine learning models based on clinical, radiology signs, and Radiomics features to predict HE. The ICH, GCS, and NIHSS scores, history of hypertension (HTN), systolic and diastolic blood pressure at the time of admission, INR, glucose level, and treatment by antiplatelets and anticoagulants were associated with moderate accuracy in predicting HE in sBGH. On the other hand, the models based on the Radiomic features performed much higher. We achieved the best performance by the Random Forest based on the 10 most predictive Radiomic features for a threshold of 25% as the definition of HE. Our study shows that using a threshold of 25% enlargement of hematoma as the definition of HE is more suitable for machine learning models. Our study is based on 3D volume measurement of hematoma volume in the range of cubic millimeters and is superior to traditional measurements based on the longest diameters on 2D or 3D images.

So far, the application of Radiomic in predicting cerebral HE is promising. Ma et al. reported an accuracy of 82% in predicting HE in hypertensive intraparenchymal hematomas [23]. Song et al. compared the “Clinical-Radiology” models and Radiomic models. They reported the Radiomic model outperformed the “clinical-radiology” models [27]. In a recent study, Radiomic analysis and quantitative satellite sign can identify the association between quantitative imaging features and hematoma pathophysiology and predict intracerebral hematoma expansion effectively and precisely in CT images [28]. However, not all studies about the role of Radiomic in HE are promising; Chen et al. have reported a moderate performance of Radiomics in HE [29]. Our results are compatible with another work [30], where authors compared Radiomics with the clinical model to predict HE. They also reported that Radiomics outperformed the clinical model [30]. We also added the radiology signs to the clinical model, and again Radiomics outperformed the “clinical+radiology sign” model. Pszczolkowski et al. have reported that the Radiomic models outperformed the “radiology-signs” model and are equal to the “clinical model” in HE prediction [31]. Based on our study, the Radiomics models outperform both “clinical” and “radiology-sign” models individually or in combination. Like our study, Xie et al. also reported that Radiomic outperformed radiology-based models for HE [32]. Their radiology-based model constituted of features regarding location, shape, density, hypodensities within hematoma, swirl sign, blend sign, black-hole sign, and island sign.

We and other prior similar studies [23,27-32] have used different datasets and totally different Radiomics pipelines (different Radiomics software, number of extracted features, number of selected features, machine learning); despite these differences in study design, our results are very similar. This suggests that the Radiomic analysis can capture enough information from the hematoma to predict HE regardless of patients’ population, scanning techniques, and Radiomics pipeline. Given this generalizability, there is a high chance of clinical application of Radiomics for HE. Moreover, we used CT images acquired on different vendor machines, and we did not use any harmonization techniques (e.g., ComBat harmonization) on the Radiomics features; hence, our model has a high performance. Again, this would indicate the generalizability of Radiomics for HE.

However, this study has several limitations. It is a pilot retrospective study on a relatively small patient population. Although the laboratory data is fairly accurate, the clinical data (e.g., ICH, GCS, and NIHHS scores) were recorded by different physicians, which can introduce variabilities in the study. To increase accuracy, all sBGHs were manually segmented slice by slice and were confirmed by the second radiologist, which is a very time-consuming approach and cannot be replicated in daily clinical practice. Only basal ganglia hematoma was evaluated for segmentation, and associated intra-ventricular bleeding was not used. Our results are focused on the basal ganglia hematoma and may not be generalized for parenchymal hemorrhages in other regions of the brain (pontine, cerebellar, and lobar hematomas). In our study, the hematoma itself was segmented; however, the perihematomal Radiomics features can also predict the chance of HE, as proposed by Zhu et al. [10]. In the future, larger prospective studies will be needed to validate the role of Radiomics in predicting HE in sBGH patients.

## Conclusions

Our pilot results show that Radiomics may outperform conventional “clinical” and “radiology sign” models in HE. Random Forest models are promising to predict HE by Radiomics. Although the application of Radiomics in HE should be tested in prospective studies, our results and previously published data are very promising.
